# Closing the gap: Contribution of surgical best practices to outcome differences between high‐ and low‐volume centers for lung cancer resection

**DOI:** 10.1002/cam4.3055

**Published:** 2020-04-21

**Authors:** Mitchell S. von Itzstein, Rong Lu, Kemp H. Kernstine, Ethan A. Halm, Shidan Wang, Yang Xie, David E. Gerber

**Affiliations:** ^1^ Department of Internal Medicine University of Texas Southwestern Medical Center Dallas TX USA; ^2^ Quantitative Biomedical Research Center University of Texas Southwestern Medical Center Dallas TX USA; ^3^ Department of Cardiothoracic Surgery University of Texas Southwestern Medical Center Dallas TX USA; ^4^ Department of Population and Data Sciences University of Texas Southwestern Medical Center Dallas TX USA; ^5^ Harold C. Simmons Comprehensive Cancer Center University of Texas Southwestern Medical Center Dallas TX USA

**Keywords:** guidelines, lobectomy, National Cancer Database (NCDB), thoracic surgery, volume‐outcome relationship

## Abstract

**Background:**

Clinical outcomes for resected early‐stage non‐small cell lung cancer (NSCLC) are superior at high‐volume facilities, but reasons for these differences remain unclear. Understanding these differences and optimizing outcomes across institutions are critical to the management of the increasing incidence of these cases. We evaluated the extent to which surgical best practices account for resected early‐stage NSCLC outcome differences between facilities according to case volume.

**Methods:**

We performed a retrospective cohort study for clinical stage 1 or 2 NSCLC undergoing surgical resection from 2004 to 2013 using the National Cancer Database (NCDB). Surgical best practices (negative surgical margins, lobar or greater resection, lymph node (LN) dissection, and examination of > 10 LNs) were compared between the highest and lowest quartile volumes.

**Results:**

A total of 150,179 patients were included in the cohort (89% white, 53% female, median age 68 years). In a multivariate model, superior overall survival (OS) was observed at highest volume centers compared to lowest volume centers (hazard ratio (HR) = 0.89; 95% CI, 0.82‐0.96; *P* = .002). After matching for surgical best practices, there was no significant OS difference (HR = 0.95; 95% CI, 0.87‐1.05; *P* = .32). Propensity score‐adjusted HR estimates indicated that surgical best practices accounted for 54% of the numerical OS difference between low‐volume and high‐volume centers. Each surgical best practice was independently associated with improved OS (all *P* ≤ .001).

**Conclusion:**

Quantifiable and potentially modifiable surgical best practices largely account for resected early‐stage NSCLC outcome differences observed between low‐ and high‐volume centers. Adherence to these guidelines may reduce and potentially eliminate these differences.

## Graphical abstract

High‐volume facilities have been observed to be associated with improved outcomes compared to low‐volume facilities for non‐small cell lung cancer surgery. We found that controlling for surgical quality measures minimizes outcome differences between facility types, suggesting that high‐quality care provision for lung cancer surgery requires compliance with surgical quality measures.

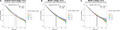

**Abbreviations**
AJCC: American Joint Commission on Cancer
ASA: American Society of Anesthesiologists
CD: Charlson‐Deyo
CoC: Commission on Cancer
CT: computed tomography
KM: Kaplan‐Meier
LN: lymph node
NCDB: National Cancer Database
NSCLC: non‐small cell lung cancer
OS: overall survival
PET: positron‐emission tomography
PUF: participant user file
TNM: tumor‐node‐metastasis

## 1 INTRODUCTION

There has been much discussion in recent decades about the relationship between facility type and volume and outcomes for non‐small cell lung cancer (NSCLC) and other malignancies, with many studies finding that institutional case volume is associated with improved surgical outcomes.^1^, ^2^, ^3^, ^4^, ^5^, ^6^, ^7^ This observation has major health‐care practice and policy implications. As the population ages and uptake of computed tomography (CT)‐based lung cancer screening increases, the number of early‐stage, potentially resectable NSCLC cases is expected to grow. If optimal care requires treatment at a limited number of high‐volume clinical centers, patients and their families may be required to travel extensively or even temporarily relocate. Such arrangements could exacerbate the financial impact of diagnosis and treatment if individuals need to pay for travel and housing, or miss additional workdays.

Case volume may serve as a proxy for multiple factors associated with improved outcomes. These may include patient differences, clinician differences, and process differences.^7^ Specifically, less sick individuals may be more likely to be referred or travel to high‐volume centers. Surgeons and other physicians achieve proficiency by performing a procedure many times. Medical centers that perform more lung cancer resections may have greater institutional memory and clinical experience—variables that are challenging to define, measure, and replicate. Additionally, high‐volume centers may be more likely to employ certain surgical and medical techniques and protocols that directly produce better risk‐adjusted outcomes.

Although many patient and clinician differences are difficult to ascertain, characterize, and control, process variables may be more readily addressed. Most widely accepted best practices for lung cancer surgery are readily defined, easily measured, and potentially feasible to benchmark across centers. Examples include the type of resection (lobar vs sublobar),^8^ surgical margin status,^9^ and the nature of lymph node (LN) examination.^10^, ^11^, ^12^ To determine the extent to which these variables may account for improved overall survival (OS) at high‐volume institutions, we examined surgical practices and clinical outcomes in a nationally representative sample, the National Cancer Database (NCDB).

## 2 MATERIALS AND METHODS

### 2.1 Data source and collection

Formed in 1989, the NCDB collects data from more than 1500 US hospitals that have been accredited by the American College of Surgeons Commission on Cancer (CoC) and the American Cancer Society, capturing an estimated 80% of newly diagnosed lung cancers in the United States.^13^, ^14^

We examined NCDB participant user files (PUF) from 2004 to 2013 for NSCLC cases. The PUF includes patients with a histological diagnosis of NSCLC (squamous cell, adenocarcinoma, sarcomatoid, adenosquamous, and other NSCLC). We identified cases with American Joint Commission on Cancer (AJCC) 8th edition^15^ clinical stage 1 or 2 NSCLC who underwent surgical resection. Cases staged per previous AJCC editions were forward‐staged as previously described^16^; those that were unable to be forward‐staged were excluded. Other histologic subtypes (carcinoid, other neuroendocrine histology, such as small cell lung cancer, and metastatic malignancies to the lung) were excluded.

We abstracted the following variables for each case: patient characteristics [age, sex, race, Hispanic origin, insurance status, income, education, Charlson‐Deyo (CD) comorbidity score (0, 1, 2, ≥3)^17^], disease characteristics [AJCC clinical stage, tumor‐node‐metastasis (TNM) edition number, primary site, laterality, histology, grade, size of tumor, and year of diagnosis], treatment characteristics [surgical margin status (positive/negative), surgical procedure of the primary site (wedge resection, segmental resection, lobectomy, and pneumonectomy), number of regional LNs examined, regional LN dissection performed (yes/no), administration of radiation therapy (yes/no), administration of chemotherapy (yes/no)], facility characteristics [location (geographic region) and total number of NSCLC stage 1‐2 surgical cases during the study period], and clinical outcome measures [last contact or death, and PUF vital status]. We defined surgical best practices as achievement of negative surgical margins, performance of lobar or greater resection, examination of >10 LNs, and performance of regional LN dissection (yes or no), consistent with current clinical guidelines.^18^, ^19^

### 2.2 Statistical analysis

We abstracted total number of lung cancer resections performed at each NCDB facility in the most recent year of analysis, as described previously,^4^ and used this metric to define annual surgical volume for each facility. We then calculated summary statistics of annual surgical resection volumes across facilities and used quartile estimates to define low‐ and high‐volume facilities. Centers in the lowest quartile were determined to be low‐volume (<6 annual NSCLC resection cases) and those in the highest quartile were deemed high‐volume (>34 annual NSCLC resection cases). For survival analyses, we defined OS as the time from definitive surgical procedure to death from any cause or last contact. Cases without a known date of death were censored at the last date of known follow‐up. Kaplan‐Meier OS curves were generated to visualize OS. Cox regression models and Wald tests were used to compare OS differences and estimate hazard ratios in both univariate and multivariate analyses. Because our sample size is sufficiently large, we excluded all records with missing data. We did not use any imputation methods in this study. To rule out the effect of potential confounders, propensity score matching was used to balance patient groups with different demographic and clinical characteristics.^20^ All variables listed in Table 1 were considered in propensity score matching to minimize the effect of collinearity. To ensure the comparability between Model 1 (propensity score matching on clinical and demographic variables) and Model 2 (propensity score matching on clinical, demographic, and surgical best practice variables), we used fixed caliper = 0.0001 and ratio = 1 for both propensity score matching processes. We included in the analyses all demographic and clinical data variables available in the NCDB considered to have potential importance in lung cancer resection outcomes. Ratio = 1 was chosen to reflect the study design of 1‐to‐1 matching. To select an appropriate caliper, we scanned a list of descending calipers to compare the stability of the matching results. We chose caliper = 0.0001 as there was no substantial difference observed when using smaller caliper values. All *P*‐values were two‐sided; results were considered significant at *P* < .05. All analyses were performed with R software, version 3.4.2.^21^ We used R packages “survival” (version 2.44‐1.1), “survminer” (version 0.4.3), and “MatchIt” (version 3.0.2).

**TABLE 1:** Patient demographics and clinical characteristics

Variable	Overall	Annual surgery volume				*P*‐value
		<6	6‐15	16‐34	>34	
Number of cases, N (%)	150 179	7027 (4.7)	17 250 (11.5)	35 839 (23.9)	90 063 (60)	
Number of hospitals, N (%)	1264	299 (23.7)	316 (25)	331 (26.2)	318 (25.2)	
Age, mean (IQR)	67.7 (61, 75)	67.7 (62, 75)	67.9 (62, 75)	67.9 (62, 75)	67.6 (61, 75)	.0004
Gender, N (%)						
Female	79 913 (53.2)	3619 (51.5)	8915 (51.7)	18 878 (52.7)	48 501 (53.9)	<.0001
Male	70 266 (46.8)	3408 (48.5)	8335 (48.3)	16 961 (47.3)	41 562 (46.1)	
Race, N (%)						
White	133 597 (89)	6129 (87.2)	15 141 (87.8)	32 235 (89.9)	80 092 (88.9)	<.0001
Black	11 859 (7.9)	648 (9.2)	1571 (9.1)	2618 (7.3)	7022 (7.8)	
Other	3680 (2.5)	227 (3.2)	465 (2.7)	847 (2.4)	2141 (2.4)	
Unknown	1043 (0.7)	23 (0.3)	73 (0.4)	139 (0.4)	808 (0.9)	
Ethnicity, N (%)						
Hispanic	3522 (2.3)	189 (2.7)	686 (4)	677 (1.9)	1970 (2.2)	<.0001
Non‐hispanic	136 583 (90.9)	6482 (92.2)	15 604 (90.5)	32 234 (89.9)	82 263 (91.3)	
Unknown	10 074 (6.7)	356 (5.1)	960 (5.6)	2928 (8.2)	5830 (6.5)	
Charlson‐Deyo comorbidity score, N (%)						
0	76 492 (50.9)	3699 (52.6)	8790 (51)	17 932 (50)	46 071 (51.2)	<.0001
1	53 193 (35.4)	2413 (34.3)	5981 (34.7)	12 747 (35.6)	32 052 (35.6)	
2	20 494 (13.6)	915 (13)	2479 (14.4)	5160 (14.4)	11 940 (13.3)	
Clinical stage, N (%)						
Stage I	115 487 (76.9)	5114 (72.8)	13 128 (76.1)	27 492 (76.7)	69 753 (77.4)	<.0001
Stage II	34 692 (23.1)	1913 (27.2)	4122 (23.9)	8347 (23.3)	20 310 (22.6)	
Laterality, N (%)						
Right	87 334 (58.2)	4010 (57.1)	10 109 (58.7)	20 861 (58.2)	52 354 (58.1)	.03
Left	61 909 (41.2)	2981 (42.4)	7009 (40.7)	14 780 (41.2)	37 139 (41.2)	
Organ is not paired	824 (0.5)	34 (0.5)	97 (0.6)	169 (0.5)	524 (0.6)	
Unknown	68 (< 0.1)	2 (< 0.1)	12 (0.1)	22 (0.1)	32 (< 0.1)	
Histology, N (%)						
Adenocarcinoma	87 622 (62.1)	3768 (56.3)	9695 (59)	20 517 (60.7)	53 642 (63.7)	<.0001
Adenosquamous	3938 (2.8)	199 (3)	455 (2.8)	1016 (3)	2268 (2.7)	
Large cell	2413 (1.7)	152 (2.3)	318 (1.9)	491 (1.5)	1452 (1.7)	
Sarcomatoid	813 (0.6)	38 (0.6)	73 (0.4)	166 (0.5)	536 (0.6)	
Squamous	41 139 (29.1)	2169 (32.4)	5113 (31.1)	10 168 (30.1)	23 689 (28.1)	
Other NSCLC	5255 (3.7)	365 (5.5)	773 (4.7)	1453 (4.3)	2664 (3.2)	
Tumor size (mm), N (%)						
≤10	12 338 (8.2)	453 (6.4)	1170 (6.8)	2668 (7.4)	8047 (8.9)	<.0001
(10, 20]	53 063 (35.3)	2287 (32.5)	5932 (34.4)	12 516 (34.9)	32 328 (35.9)	
(20, 30]	40 210 (26.8)	1953 (27.8)	4866 (28.2)	9893 (27.6)	23 498 (26.1)	
(30, 40]	22 891 (15.2)	1156 (16.5)	2699 (15.6)	5480 (15.3)	13 556 (15.1)	
(40, 50]	12 138 (8.1)	655 (9.3)	1483 (8.6)	3002 (8.4)	6998 (7.8)	
(50, 70]	9539 (6.4)	523 (7.4)	1100 (6.4)	2280 (6.4)	5636 (6.3)	
Tumor grade, N (%)						
1	24 198 (16.1)	935 (13.3)	2511 (14.6)	5665 (15.8)	15 087 (16.8)	<.0001
2	64 468 (42.9)	2849 (40.5)	7526 (43.6)	15 404 (43)	38 689 (43)	
3	47 937 (31.9)	2432 (34.6)	5519 (32)	11 728 (32.7)	28 258 (31.4)	
4	2402 (1.6)	149 (2.1)	312 (1.8)	570 (1.6)	1371 (1.5)	
Unknown	11 174 (7.4)	662 (9.4)	1382 (8)	2472 (6.9)	6658 (7.4)	
Radiation therapy, N (%)						
Yes	10 936 (7.3)	739 (10.5)	1396 (8.1)	2743 (7.7)	6058 (6.7)	<.0001
No	137 055 (91.3)	6135 (87.3)	15 511 (89.9)	32 496 (90.7)	82 913 (92.1)	
Unknown	2188 (1.5)	153 (2.2)	343 (2)	600 (1.7)	1092 (1.2)	
Chemotherapy, N (%)						
Yes	28 631 (19.1)	1663 (23.7)	3557 (20.6)	6913 (19.3)	16 498 (18.3)	<.0001
No	116 086 (77.3)	5023 (71.5)	12 958 (75.1)	27 596 (77)	70 509 (78.3)	
Unknown	5462 (3.6)	341 (4.9)	735 (4.3)	1330 (3.7)	3056 (3.4)	
Facility location, N (%)						
New England	8512 (5.7)	375 (5.3)	1152 (6.7)	2567 (7.2)	4418 (4.9)	<.0001
Middle Atlantic	25 048 (16.7)	1003 (14.3)	2402 (13.9)	4401 (12.3)	17 242 (19.1)	
South Atlantic	33 475 (22.3)	1618 (23)	2283 (13.2)	7274 (20.3)	22 300 (24.8)	
East North Central	27 963 (18.6)	1265 (18)	4477 (26)	7228 (20.2)	14 993 (16.6)	
East South Central	13 494 (9)	388 (5.5)	962 (5.6)	2628 (7.3)	9516 (10.6)	
West North Central	11 784 (7.8)	449 (6.4)	1064 (6.2)	3415 (9.5)	6856 (7.6)	
West South Central	9384 (6.2)	446 (6.3)	1999 (11.6)	2376 (6.6)	4563 (5.1)	
Mountain	5380 (3.6)	454 (6.5)	740 (4.3)	1936 (5.4)	2250 (2.5)	
Pacific	14 123 (9.4)	989 (14.1)	2080 (12.1)	3815 (10.6)	7239 (8)	
Unknown	1016 (0.7)	40 (0.6)	91 (0.5)	199 (0.6)	686 (0.8)	
Income, N (%)						
<$30k	18 565 (12.4)	966 (13.7)	2128 (12.3)	4629 (12.9)	10 842 (12)	<.0001
[$30k, $35k)	26 995 (18)	1479 (21)	3065 (17.8)	6747 (18.8)	15 704 (17.4)	
[$35k, $46k)	41 103 (27.4)	1898 (27)	4917 (28.5)	10 053 (28.1)	24 235 (26.9)	
≥$46k	57 881 (38.5)	2388 (34)	6434 (37.3)	13 010 (36.3)	36 049 (40)	
Unknown	5635 (3.8)	296 (4.2)	706 (4.1)	1400 (3.9)	3233 (3.6)	
Education, N (%)						
<20% did not graduate high school in zip	86 727 (57.7)	3750 (53.4)	9425 (54.6)	21 157 (59)	52 395 (58.2)	<.0001
≥20% did not graduate high school in zip	57 801 (38.5)	2981 (42.4)	7116 (41.3)	13 279 (37.1)	34 425 (38.2)	
Unknown	5651 (3.8)	296 (4.2)	709 (4.1)	1403 (3.9)	3243 (3.6)	
Insurance status, N (%)						
Not insured	2542 (1.7)	157 (2.2)	353 (2)	724 (2)	1308 (1.5)	<.0001
Private insurance	45 583 (30.4)	1980 (28.2)	4854 (28.1)	10 645 (29.7)	28 104 (31.2)	
Medicaid	6089 (4.1)	368 (5.2)	813 (4.7)	1371 (3.8)	3537 (3.9)	
Medicare	92 766 (61.8)	4349 (61.9)	10 798 (62.6)	22 169 (61.9)	55 450 (61.6)	
Other government	1333 (0.9)	57 (0.8)	149 (0.9)	324 (0.9)	803 (0.9)	
Unknown	1866 (1.2)	116 (1.7)	283 (1.6)	606 (1.7)	861 (1)	
Surgical procedure, N (%)						
Sublobar resection	34 501 (23)	1618 (23)	3878 (22.5)	7983 (22.3)	21 022 (23.3)	<.0001
Lobar resection or greater	114 951 (76.5)	5348 (76.1)	13 262 (76.9)	27 728 (77.4)	68 613 (76.2)	
Unknown	727 (0.5)	61 (0.9)	110 (0.6)	128 (0.4)	428 (0.5)	
Regional lymph nodes examined, N (%)						
<10	95 285 (63.4)	4785 (68.1)	12 131 (70.3)	24 232 (67.6)	54 137 (60.1)	<.0001
≥10	44 270 (29.5)	1625 (23.1)	4136 (24)	9203 (25.7)	29 306 (32.5)	
Unknown	10 624 (7.1)	617 (8.8)	983 (5.7)	2404 (6.7)	6620 (7.4)	
Regional lymph node dissection, N (%)						
Performed	131 239 (87.4)	5836 (83.1)	14 608 (84.7)	31 001 (86.5)	79 794 (88.6)	<.0001
Not performed	18 263 (12.2)	1075 (15.3)	2510 (14.6)	4665 (13)	10 013 (11.1)	
Unknown	677 (0.5)	116 (1.7)	132 (0.8)	173 (0.5)	256 (0.3)	
Surgical margins, N (%)						
Positive	6283 (4.2)	394 (5.6)	794 (4.6)	1659 (4.6)	3436 (3.8)	<.0001
Negative	141 441 (94.2)	6383 (90.8)	16 065 (93.1)	33 610 (93.8)	85 383 (94.8)	
Unknown	2455 (1.6)	250 (3.6)	391 (2.3)	570 (1.6)	1244 (1.4)	

## 3 RESULTS

### 3.1 Demographics, clinical characteristics, facility characteristics, and surgical best practices

**FIGURE 1:**
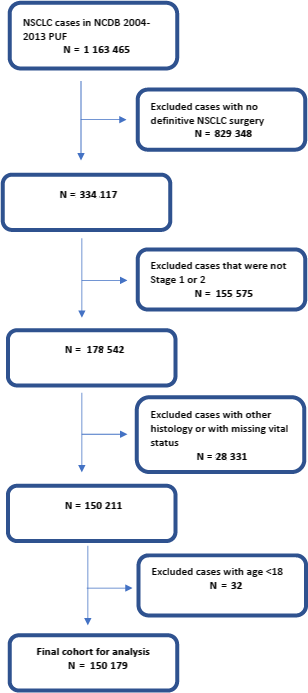
Flow diagram of patient selection with inclusion and exclusion criteria

From NCDB PUF years 2004‐2013, we identified an initial cohort of 1,163,465 NSCLC cases. We then limited our study sample to AJCC 8th edition clinical stage 1 or 2 NSCLC that underwent surgical resection, resulting in a study sample of 150,179 (12.9%) cases treated at 1,264 hospitals (Figure 1). Median age was 68 years, 89% were white, and 53% were female. Across institutions, median annual volume of stage 1 or 2 NSCLC surgical resections in 2013 was 16.

All demographic and clinical characteristics differed significantly according to facility surgical volume (Table 1). The highest and lowest volume institutions performed more sublobar resections compared to other centers. High‐volume institutions were more likely to examine greater than 10 LN, perform LN dissection, and report negative surgical margins.

### 3.2 Clinical outcomes

In univariate analysis, using the lowest volume quartile as reference, we observed further improvements in outcomes with each increase in facility case volume (Table 2). These trends were observed in the overall study cohort as well as stage 1 and 2 subgroups. In the overall cohort and stage 1 subgroup, after base matching for 16 clinical and demographic confounders (Model 1), OS was statistically equivalent in the first, second, and third quartiles across stages, but remained superior in fourth quartile. For stage 2 NSCLC, there was no significant difference in OS across quartiles after confounder matching (Model 1). After controlling for surgical best practices (Model 2), numerical differences in OS were further reduced. There was no significant difference in OS across cohorts in the overall cohort or stage 2 subgroup. In the stage 1 subgroup, only the highest quartile institutions had improved OS.

**TABLE 2:** Hazard ratio estimates prior to propensity matching (original cohort), after matching for case characteristics (Model 1), and after further matching for surgical best practices (Model 2)

Cohort	Annual surgery volume (ref: <6)	Overall		Stage I		Stage II	
		HR (95% CI)	*P*‐value	HR (95% CI)	*P*‐value	HR (95% CI)	*P*‐value
Original Cohort (N = 150,179)	6‐15	0.92 (0.88, 0.96)	.0002	0.94 (0.89, 0.99)	.01	0.93 (0.86, 1.00)	.06
	16‐34	0.90 (0.86, 0.93)	<.0001	0.91 (0.86, 0.95)	<.0001	0.93 (0.87, 1.00)	.06
	>34	0.81 (0.78, 0.84)	<.0001	0.81 (0.78, 0.85)	<.0001	0.86 (0.80, 0.91)	<.0001
Model 1^a^ (N = 16,572)	6‐15	0.96 (0.89, 1.04)	.31	0.93 (0.85, 1.02)	.13	0.98 (0.84, 1.15)	.85
	16‐34	0.95 (0.88, 1.02)	.17	0.92 (0.84, 1.00)	.05	0.98 (0.84, 1.13)	.75
	>34	0.89 (0.82, 0.96)	.002	0.84 (0.77, 0.92)	.0001	0.93 (0.81, 1.07)	.31
Model 2^a^ (N = 12,498)	6‐15	0.97 (0.87, 1.07)	.54	0.93 (0.84, 1.04)	.23	1.02 (0.74, 1.42)	.89
	16‐34	0.97 (0.88, 1.07)	.50	0.91 (0.82, 1.01)	.08	1.05 (0.77, 1.43)	.77
	>34	0.95 (0.87, 1.05)	.31	0.88 (0.80, 0.98)	.02	0.96 (0.71, 1.30)	.78

^a^ To ensure comparability between Model 1 (propensity score matching on clinical and demographic variables) and Model 2 (propensity score matching on clinical, demographic, and surgical best practice variables), we used fixed caliper = 0.0001 and ratio = 1 for both propensity score matching processes.

In multivariate analysis, each of the examined surgical best practices independently impacted OS. In the overall cohort, improved OS was observed in cases with negative surgical margins (HR 0.51; 95% CI 0.46‐0.56; *P* < .001), cases undergoing lobar or greater resection (HR 0.77; 95% CI 0.73‐0.81; *P* < .001), cases undergoing regional LN dissection (HR 0.69; 95% CI 0.65‐0.74; *P* < .001), and cases including examination of >10 LNs, as recommended by the CoC guidelines,^18^ (HR 0.90; 95% CI 0.85‐0.91; *P* < .001). Similar effects were noted in the stage 1 and stage 2 subgroups (Table 3).

**TABLE 3:** Hazard ratio estimates for effect of each surgical best practice variable before and after matching for case characteristics

Cohort	Surgical best practice variable	Overall		Stage I		Stage II	
		HR (95% CI)	*P*‐value	HR (95% CI)	*P*‐value	HR (95% CI)	*P*‐value
Original Cohort ( N = 150,179)	Surgical margins (negative vs positive)	0.50 (0.48, 0.51)	<.0001	0.51 (0.48, 0.53)	<.0001	0.61 (0.58, 0.64)	<.0001
	Surgical procedure (lobar resection or greater vs sublobar resection)	0.77 (0.76, 0.79)	<.0001	0.70 (0.68, 0.71)	<.0001	0.70 (0.67, 0.73)	<.0001
	Regional lymph nodes dissection (performed vs not performed)	0.70 (0.69, 0.72)	<.0001	0.64 (0.63, 0.66)	<.0001	0.62 (0.58, 0.66)	<.0001
	Regional lymph nodes examined (≥10 vs <10)	0.89 (0.87, 0.91)	<.0001	0.82 (0.80, 0.84)	<.0001	0.86 (0.84, 0.89)	<.0001
Model 1^a^ (N = 16,572)	Surgical margins (negative vs positive)	0.51 (0.46, 0.56)	<.0001	0.53 (0.46, 0.60)	<.0001	0.59 (0.52, 0.68)	<.0001
	Surgical procedure (lobar resection or greater vs sublobar resection)	0.77 (0.73, 0.81)	<.0001	0.70 (0.66, 0.74)	<.0001	0.67 ( 0.58,0.76)	<.0001
	Regional lymph nodes dissection (performed vs not performed)	0.69 (0.65, 0.74)	<.0001	0.64 (0.60, 0.69)	<.0001	0.51 (0.43, 0.60)	<.0001
	Regional lymph nodes examined (≥10 vs <10)	0.90 (0.85, 0.95)	.0002	0.85 (0.79, 0.91)	<.0001	0.82 (0.75, 0.90)	<.0001

^a^ This is the cohort matched on clinical and demographic variables between surgery volume groups, using propensity score with caliper = 0.0001 and ratio = 1.

We compared HRs with and without best practice propensity matching to numerically estimate the influence these variables have on outcome differences, with hazard ratio 1 considered as equivalent outcome, determined as follows: surgical best practices influence = ((1 − HR_1_) − (1 − HR_2_))/(1 − HR_1_) × 100. Using this approach, in the overall cohort after multivariate matching, surgical best practices accounted for 54% of the numerical OS difference between the lowest volume compared to the highest volume centers.

**FIGURE 2:**
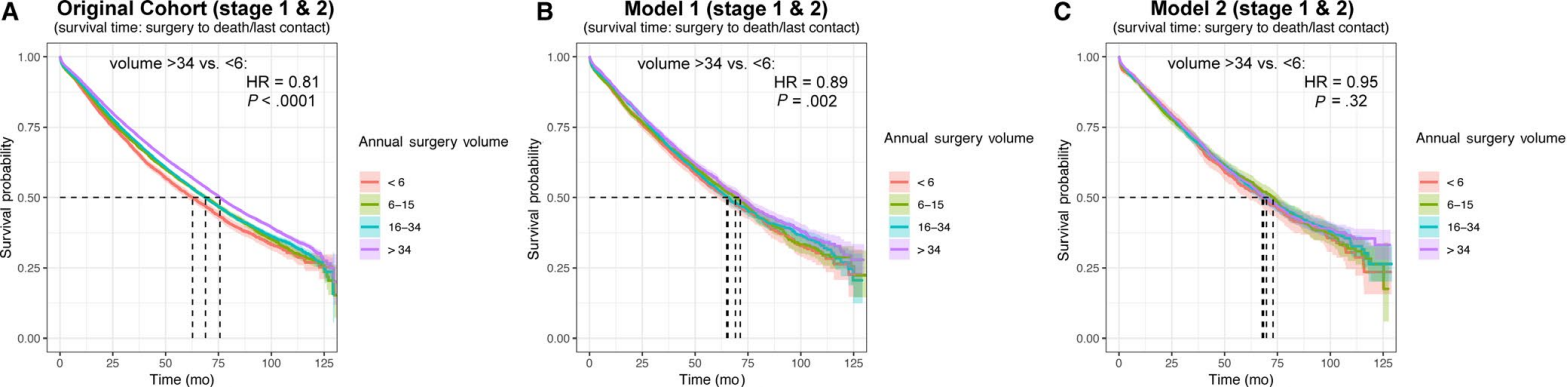
Kaplan‐Meier overall survival for overall cohort prior to propensity matching (original cohort), after matching for case characteristics (Model 1), and after further matching for surgical best practices (Model 2)

**FIGURE 3:**
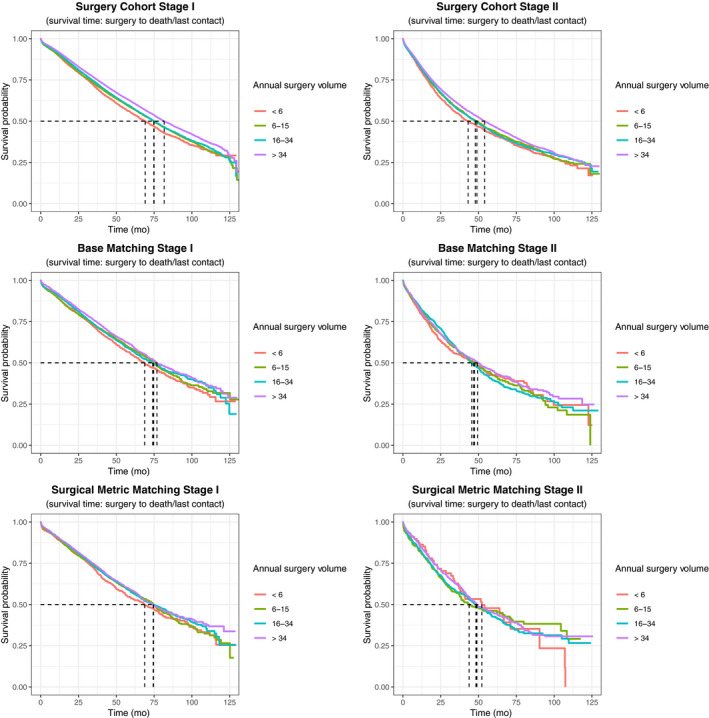
Kaplan‐Meier overall survival for stage 1 and stage 2 cohort prior to propensity matching (original cohort), after matching for case characteristics (Model 1), and after further matching for surgical best practices (Model 2)

Figure 2 shows Kaplan‐Meier plots of OS for the overall cohort between facility volume quartiles for univariate (Panel A), multivariate (Model 1, Panel B), and surgical best practice‐matched (Model 2, Panel C) cohorts. *P* values and hazard ratios are propensity score‐adjusted for the matched cohorts. Subgroup analyses for stages 1 and 2 are shown in Figure 3

## 4 DISCUSSION

For decades, it has been observed that high‐volume centers have improved surgical outcomes for early‐stage NSCLC. In the present study, we sought to identify specific and potentially modifiable factors accounting for these differences. In this national cohort of more than 150 000 patients with surgically resected clinical stage 1‐2 NSCLC treated at more than 1200 facilities, we again noted that OS was superior at high‐volume centers, even after adjusting for more than one dozen demographic and clinical factors. As noted in earlier landmark studies,^4^ the greatest outcome differences occurred between the lowest‐ and highest volume centers, and the current study demonstrated a comparable trend for volume‐outcome association for OS. However, when we incorporated surgical best practices into the analysis, the magnitude of these outcome differences declined substantially and no longer had statistical significance. Indeed, OS curves were essentially overlapping. These findings suggest that greater dissemination of and adherence to practice guidelines may largely close the outcome gap between large‐ and small‐volume facilities.

In this study, we selected surgical best practices that are widely endorsed,^22^ readily recorded and assessed, and have the potential for widespread implementation and benchmarking for quality improvement: type of resection, LN examination, and surgical margin status. As previously shown,^9^, ^22^ each of these variables was associated with clinical outcomes in this study including a previous analysis demonstrating that combining surgical quality measures improves OS.^23^ Among them, surgical margin status had the strongest association, with a 50% reduction in the risk of death for cases with negative margins. Regional LN dissection was associated with a 30% reduction in the risk of death, while lobar or greater (anatomical) resection was associated with a 25% reduction in the risk of death. High‐volume clinical centers were more likely to achieve negative surgical margins and to perform an adequate LN dissection.

Each surgical best practice was independently statistically and clinically important across the overall cohort and both stage 1 and 2 subgroups. This is expected and consistent with previous studies, and reinforces the importance and appropriateness of guideline‐directed care for NSCLC resections. Interestingly, performance of LN dissection was most strongly associated with improved OS for the stage 2 cohort, being associated with a 50% reduction in mortality. This finding could be related to the removal of occult metastasis with LN dissection or upstaging and appropriate treatment of LN disease when discovered.

Importantly, it seems feasible to benchmark and export these metrics to improve surgical outcomes across centers. For example, both provision of a surgical LN specimen collection kit and a novel, more thorough pathologic gross dissection method have been shown to significantly improve rates of adequate LN examination independently and when performed together.^24^, ^25^ It has also been shown that multidisciplinary lung cancer care can be implemented in a community health‐care setting.^26^ Nevertheless, other recommendations may be more difficult to define and thus more challenging to transmit. The achievement of negative surgical margins could reflect tumor location and other attributes, and does not reflect an a priori decision such as LN dissection or resection type. Furthermore, although radiation therapy for positive margins is generally recommended as a treatment option for cases with positive margins, it has not been validated in population analyses.^27^

Even after adjusting for surgical best practices, a modest but significant OS benefit persisted for stage 1 cases. The precise reasons for this observation are not clear. Stage 1 lung cancer represents a widely heterogeneous population, ranging from poorly differentiated, invasive cancers that likely have distant micrometastatic disease at diagnosis to incidentally detected, small, non‐ or minimally invasive tumors that might never impact patient quantity or quality of life even if left untreated. One possibility is that some clinical stage 2 cases derived OS benefit from removal of occult LN metastasis that was not present in clinical stage 1 cases, leading to increased effect of surgical quality measures. It is also possible that stage 1 cases at high‐volume centers were more likely to represent particularly low‐risk (based on size and/or histology) tumors. These facilities performed a greater proportion of sublobar resections, which are recommended by numerous expert guidelines for cases such as pure ground‐glass opacities or adenocarcinoma in situ under 2 cm.^19^, ^28^, ^29^, ^30^ Notably, sublobar resection was also performed more frequently at the lowest volume centers. We are unable to determine whether sublobar resections were performed (a) following guidance for the lowest risk tumors, (b) because the patient was not a candidate for lobectomy, or (c) because the treatment team was unaware of surgical best practices.

One previously proposed strategy to improve surgical outcomes is to limit who performs procedures. For NSCLC, it has been suggested that complex resections be performed only by individuals and facilities meeting minimal annual volume thresholds, specifically 20 per surgeon and 40 per facility.^31^ The facility cut‐off suggestion is consistent with our study finding that the highest quartile of facilities perform >34 annual NSCLC resections, a threshold comparable to that of high‐volume centers in earlier studies.^4^ Another recent analysis revealed that patients undergoing resection at top‐ranked cancer centers have better postoperative outcomes compared to those receiving care at their affiliated centers.^32^ In that study, there were large mean volume differences between affiliated (8 cases/year) and top‐ranked (77 cases/year) facilities. The current analysis suggests that clinically meaningful outcome differences could potentially be minimized by promoting guideline‐directed surgical care, rather than restricting access to only high‐volume surgeons and facilities. A recent analysis of stage IIIA NSCLC found that in these cases, patients being treated at high‐volume facilities were more likely to receive surgical resection and had improved OS compared to low‐volume facilities.^33^ Interestingly, another recent analysis revealed the improved OS trend at high‐volume facilities persisted for an analysis of stage IV NSCLC, suggesting that factors other than surgical techniques are involved.^34^

A limitation of this study is the nature of available clinical data. While the NCDB provides extensive data on NSCLC cases, several variables relevant to clinical outcomes are not collected. These include the American Society of Anesthesiologists (ASA) physical status classification, smoking status, pulmonary function, weight, body mass index, performance status, and living arrangement.^5^, ^13^ Surgeon type and case volume were also not available, both of which may be associated with complications and mortality rates.^7^, ^35^, ^36^ Nor does the NCDB examine other factors that may influence outcomes, such as preoperative positron‐emission tomography (PET)/CT, brain imaging, and bronchoscopy.^37^ Some patients may have had comorbidities that precluded lobectomy, and therefore sublobar resection may have represented best surgical practice in those cases. Charlson comorbidity score propensity matching and the finding that the rates of lobar or greater resection were comparable between the lowest and highest facilities make this limitation less important to study outcomes. It is also possible that surgical best practices could represent surrogate markers for one of the above variables that are known to influence outcomes or other unknown variables, for example other unmeasured surgical techniques or quality of pathological examination. Although the current analysis does not provide details of surgical technique, such as video‐assisted or robotic, these approaches have been shown to yield comparable survival to thoracotomy and therefore may not alter our findings.^6^, ^38^, ^39^

In conclusion, we have found that modifiable surgical best practices account for a meaningful proportion of outcome differences between high‐ and low‐volume centers for resectable early‐stage NSCLC. One response to these findings is to consolidate early‐stage NSCLC surgical treatment at selected high‐volume facilities, as has been suggested.^31^ However, growing case numbers, geographic distribution, and patient preferences and circumstances may not permit such an approach in many cases. For instance, smoking rates and lung cancer diagnoses are generally higher in rural areas,^40^, ^41^ which are less likely to have high‐volume clinical centers. Because it may not always be practical to consolidate treatment of the growing number of early‐stage NSCLC at select sites nationwide, it seems reasonable to continue and expand efforts to promote best practices across centers to minimize outcome disparities.

## CONFLICT OF INTERESTS

The authors report no conflict of interest.

## AUTHOR CONTRIBUTIONS

DEG, MVI, RL: Conceptualization and methodology; MVI: Original draft; YX, RL: Data curation and formal analysis; DEG, RL, KHK, EAH, SW, YX: Interpretation, review, and revisions; DEG, YX: Supervision and resources.

## Data Availability

The data used in the study are derived from a de‐identified NCDB file. These data may be obtained via request to the NCDB.

## References

[1] Cheung MC , Hamilton K , Sherman R , et al. Impact of teaching facility status and high‐volume centers on outcomes for lung cancer resection: an examination of 13,469 surgical patients. Ann Surg Oncol. 2009;16(1):3‐13. 18600379 10.1245/s10434-008-0025-9

[2] Liu JB , Bilimoria KY , Mallin K , Winchester DP . Patient characteristics associated with undergoing cancer operations at low‐volume hospitals. Surgery. 2017;161(2):433‐443. 27590617 10.1016/j.surg.2016.07.027

[3] Birkmeyer JD , Sun Y , Wong SL , Stukel TA . Hospital volume and late survival after cancer surgery. Ann Surg. 2007;245(5):777‐783. 17457171 10.1097/01.sla.0000252402.33814.dd PMC1877074

[4] Bach PB , Cramer LD , Schrag D , Downey RJ , Gelfand SE , Begg CB . The influence of hospital volume on survival after resection for lung cancer. N Engl J Med. 2001;345(3):181‐188. 11463014 10.1056/NEJM200107193450306

[5] Rosen JE , Hancock JG , Kim AW , Detterbeck FC , Boffa DJ . Predictors of mortality after surgical management of lung cancer in the National Cancer Database. Ann Thorac Surg. 2014;98(6):1953‐1960. 25443003 10.1016/j.athoracsur.2014.07.007

[6] Numan RC , Berge MT , Burgers JA , et al. Peri‐ and postoperative management of stage I‐III non small cell lung cancer: which quality of care indicators are evidence‐based? Lung Cancer. 2016;101:129‐136. 27794401 10.1016/j.lungcan.2016.06.007

[7] Halm EA , Lee C , Chassin MR . Is volume related to outcome in health care? A systematic review and methodologic critique of the literature. Ann Intern Med. 2002;137(6):511‐520. 12230353 10.7326/0003-4819-137-6-200209170-00012

[8] Hattori A , Matsunaga T , Takamochi K , Oh S , Suzuki K . Locoregional recurrence after segmentectomy for clinical‐T1aN0M0 radiologically solid non‐small‐cell lung carcinoma. Eur J Cardiothorac Surg. 2017;51(3):518‐525. 28082475 10.1093/ejcts/ezw336

[9] Lin CC , Smeltzer MP , Jemal A , Osarogiagbon RU . Risk‐adjusted margin positivity rate as a surgical quality metric for non‐small cell lung cancer. Ann Thorac Surg. 2017;104(4):1161‐1170. 28709665 10.1016/j.athoracsur.2017.04.033 PMC5610071

[10] Shen‐Tu Y , Mao F , Pan Y , et al. Lymph node dissection and survival in patients with early stage nonsmall cell lung cancer: a 10‐year cohort study. Medicine (Baltimore). 2017;96(43):e8356. 29069017 10.1097/MD.0000000000008356 PMC5671850

[11] Zhou H , Tapias LF , Gaissert HA , et al. Lymph node assessment and impact on survival in video‐assisted thoracoscopic lobectomy or segmentectomy. Ann Thorac Surg. 2015;100(3):910‐916. 26165483 10.1016/j.athoracsur.2015.04.034

[12] Stiles BM , Kamel MK , Nasar A , et al. The importance of lymph node dissection accompanying wedge resection for clinical stage IA lung cancer. Eur J Cardiothorac Surg. 2017;51(3):511‐517. 28007869 10.1093/ejcts/ezw343

[13] Boffa DJ , Rosen JE , Mallin K , et al. Using the national cancer database for outcomes research: a review. JAMA Oncol. 2017;3(12):1722‐1728. 28241198 10.1001/jamaoncol.2016.6905

[14] Bilimoria KY , Stewart AK , Winchester DP , Ko CY . The National Cancer Data Base: a powerful initiative to improve cancer care in the United States. Ann Surg Oncol. 2008;15(3):683‐690. 18183467 10.1245/s10434-007-9747-3 PMC2234447

[15] Lim W , Ridge CA , Nicholson AG , Mirsadraee S . The 8(th) lung cancer TNM classification and clinical staging system: review of the changes and clinical implications. Quant Imaging Med Surg. 2018;8(7):709‐718. 30211037 10.21037/qims.2018.08.02 PMC6127520

[16] Yang L , Wang S , Zhou Y , et al. Evaluation of the 7(th) and 8(th) editions of the AJCC/UICC TNM staging systems for lung cancer in a large North American cohort. Oncotarget. 2017;8(40):66784‐66795. 28977996 10.18632/oncotarget.18158 PMC5620136

[17] Deyo RA , Cherkin DC , Ciol MA . Adapting a clinical comorbidity index for use with ICD‐9‐CM administrative databases. J Clin Epidemiol. 1992;45(6):613‐619. 1607900 10.1016/0895-4356(92)90133-8

[18] Cancer Co. Cancer Programs Practice Profile Reports (CP3R): Lung measure specifications. 2018 https://www.facs.org/~/media/files/quality%20programs/cancer/ncdb/measure%20specs%20nscl.ashx. Accessed November 20, 2019.

[19] National Comprehensive Cancer N. Non‐small cell lung cancer 2019 https://www.nccn.org/professionals/physician_gls/pdf/nscl_blocks.pdf. Accessed November 20, 2019.

[20] Burgette L , Griffin BA , McCaffrey D . Propensity scores for multiple treatments: a tutorial for the mnps function in the twang package R package Rand Corporation. 2017;478.

[21] Team RC . R: A language adn environment for statistial computing. Vienna, Austria: R Foundation for Statistical Computing; 2016.

[22] Osarogiagbon RU , Ray MA , Faris NR , et al. Prognostic value of national comprehensive cancer network lung cancer resection quality criteria. Ann Thorac Surg. 2017;103(5):1557‐1565. 28366464 10.1016/j.athoracsur.2017.01.098 PMC5401641

[23] Samson P , Crabtree T , Broderick S , et al. Quality measures in clinical stage I non‐small cell lung cancer: improved performance is associated with improved survival. Ann Thorac Surg. 2017;103(1):303‐311. 27665480 10.1016/j.athoracsur.2016.07.003 PMC5182109

[24] Ray MA , Faris NR , Smeltzer MP , et al. Effectiveness of implemented interventions on pathologic nodal staging of non‐small cell lung cancer. Ann Thorac Surg. 2018;106(1):228‐234. 29534956 10.1016/j.athoracsur.2018.02.021 PMC6019187

[25] Heldwein M , Michel M , Doerr F , Hekmat K . Meticulous lymph node dissection and gross pathological examination improves survival in non‐small cell lung cancer patients. J Thorac Dis. 2018;10(Suppl 33):S3951‐S3953. 30631524 10.21037/jtd.2018.09.53 PMC6297549

[26] Smeltzer MP , Rugless FE , Jackson BM , et al. Pragmatic trial of a multidisciplinary lung cancer care model in a community healthcare setting: study design, implementation evaluation, and baseline clinical results. Transl Lung Cancer Res. 2018;7(1):88‐102. 29535915 10.21037/tlcr.2018.01.02 PMC5835591

[27] Smeltzer MP , Lin CC , Kong FS , Jemal A , Osarogiagbon RU . Survival impact of postoperative therapy modalities according to margin status in non‐small cell lung cancer patients in the United States. J Thorac Cardiovasc Surg. 2017;154(2):661‐672.e10. 28483267 10.1016/j.jtcvs.2017.03.085 PMC5519432

[28] Postmus PE , Kerr KM , Oudkerk M , et al. Early and locally advanced non‐small‐cell lung cancer (NSCLC): ESMO Clinical Practice Guidelines for diagnosis, treatment and follow‐up. Ann Oncol. 2017;28:iv1‐iv21. 10.1093/annonc/mdx222 28881918

[29] Ramnath N , Dilling TJ , Harris LJ , et al. Treatment of stage III non‐small cell lung cancer: diagnosis and management of lung cancer, 3rd ed: American College of Chest Physicians evidence‐based clinical practice guidelines. Chest. 2013;143(5 Suppl):e314S‐e340. 23649445 10.1378/chest.12-2360

[30] Howington JA , Blum MG , Chang AC , Balekian AA , Murthy SC . Treatment of stage I and II non‐small cell lung cancer: Diagnosis and management of lung cancer, 3rd ed: American College of Chest Physicians evidence‐based clinical practice guidelines. Chest. 2013;143(5 Suppl):e278S‐e313S. 23649443 10.1378/chest.12-2359

[31] Urbach DR . Pledging to eliminate low‐volume surgery. N Engl J Med. 2015;373(15):1388‐1390. 26444728 10.1056/NEJMp1508472

[32] Hoag JR , Resio BJ , Monsalve AF , et al. Differential safety between top‐ranked cancer hospitals and their affiliates for complex cancer surgery. JAMA Netw Open. 2019;2(4):e191912. 30977848 10.1001/jamanetworkopen.2019.1912 PMC6481444

[33] Kommalapati A , Tella SH , Appiah AK , Smith L , Ganti AK . Association between treatment facility volume, therapy types, and overall survival in patients with stage IIIA non‐small cell lung cancer. J Natl Compr Canc Netw. 2019;17(3):229‐236. 30865920 10.6004/jnccn.2018.7086

[34] Goyal G , Kommalapati A , Bartley AC , Gunderson TM , Adjei AA , Go RS . Association between hospital volume and mortality of patients with metastatic non‐small cell lung cancer. Lung Cancer. 2018;122:214‐219. 30032834 10.1016/j.lungcan.2018.06.025

[35] Smith CB , Wolf A , Mhango G , Wisnivesky JP . Impact of surgeon volume on outcomes of older stage I lung cancer patients treated via video‐assisted thoracoscopic surgery. Semin Thorac Cardiovasc Surg. 2017;29(2):223‐230. 28823334 10.1053/j.semtcvs.2017.01.013

[36] Goodney PP , Lucas FL , Stukel TA , Birkmeyer JD . Surgeon specialty and operative mortality with lung resection. Ann Surg. 2005;241(1):179‐184. 15622006 10.1097/01.sla.0000149428.17238.03 PMC1356861

[37] Smeltzer MP , Faris NR , Ray MA , et al. Survival before and after direct surgical quality feedback in a population‐based lung cancer cohort. Ann Thorac Surg. 2019;107(5):1487‐1493. 30594579 10.1016/j.athoracsur.2018.11.058 PMC6478525

[38] Louie BE , Wilson JL , Kim S , et al. Comparison of video‐assisted thoracoscopic surgery and robotic approaches for clinical stage I and stage II non‐small cell lung cancer using the society of thoracic surgeons database. Ann Thorac Surg. 2016;102(3):917‐924. 27209613 10.1016/j.athoracsur.2016.03.032 PMC5198574

[39] Yang H‐X , Woo KM , Sima CS , et al. Long‐term survival based on the surgical approach to lobectomy for clinical stage I nonsmall cell lung cancer: comparison of robotic, video‐assisted thoracic surgery, and thoracotomy lobectomy. Ann Surg. 2017;265(2):431‐437. 28059973 10.1097/SLA.0000000000001708 PMC5033685

[40] Jenkins WD , Matthews AK , Bailey A , et al. Rural areas are disproportionately impacted by smoking and lung cancer. Prev Med Rep. 2018;10:200‐203. 29868368 10.1016/j.pmedr.2018.03.011 PMC5984228

[41] Atkins GT , Kim T , Munson J . Residence in rural areas of the United States and lung cancer mortality. disease incidence, treatment disparities, and stage‐specific survival. Ann Am Thorac Soc. 2017;14(3):403‐411. 28118039 10.1513/AnnalsATS.201606-469OC

